# Probabilistic inference and ranking of gene regulatory pathways as a shortest-path problem

**DOI:** 10.1186/1471-2105-14-S13-S5

**Published:** 2013-10-01

**Authors:** James D Jensen, Daniel M Jensen, Mark J Clement, Quinn O Snell

**Affiliations:** 1 Dept. of Bioinformatics and Systems Biology, University of California, San Diego, La Jolla, CA, USA; 2 Department of Mathematics, Brigham Young University, Provo, Utah, USA; 3 Department of Computer Science, Brigham Young University, Provo, Utah, USA

## Abstract

**Background:**

Since the advent of microarray technology, numerous methods have been devised to infer gene regulatory relationships from gene expression data. Many approaches that infer entire regulatory networks. This produces results that are rich in information and yet so complex that they are often of limited usefulness for researchers. One alternative unit of regulatory interactions is a linear path between genes. Linear paths are more comprehensible than networks and still contain important information. Such paths can be extracted from inferred regulatory networks or inferred directly. Since criteria for inferring networks generally differs from criteria for inferring paths, indirect and direct inference of paths may achieve different results.

**Results:**

This paper explores a strategy to infer linear pathways by converting the path inference problem into a shortest-path problem. The edge weights used are the negative log-transformed probabilities of directness derived from the posterior joint distributions of pairwise mutual information between gene expression levels. Directness is inferred using the data processing inequality. The method was designed with two goals. One is to achieve better accuracy in path inference than extraction of paths from inferred networks. The other is to facilitate priorization of interactions for laboratory validation. A method is proposed for achieving this by ranking paths according to the joint probability of directness of each path's edges. The algorithm is evaluated using simulated expression data and is compared to extraction of shortest paths from networks inferred by two alternative methods, ARACNe and a minimum spanning tree algorithm.

**Conclusions:**

Direct path inference appears to achieve accuracy competitive with that obtained by extracting paths from networks inferred by the other methods. Preliminary exploration of the use of joint edge probabilities to rank paths is largely inconclusive. Suggestions for a better framework for such comparisons are discussed.

## Background

Gene microarrays and RNA-seq both measure the simultaneous expression (i.e. amount of RNA transcript) of hundreds or thousands of genes. The relationship between genes' expression levels across multiple samples can be used to infer regulatory relationships. Inferring these relationships computationally can focus research and save time and expense.

**Figure 1:**
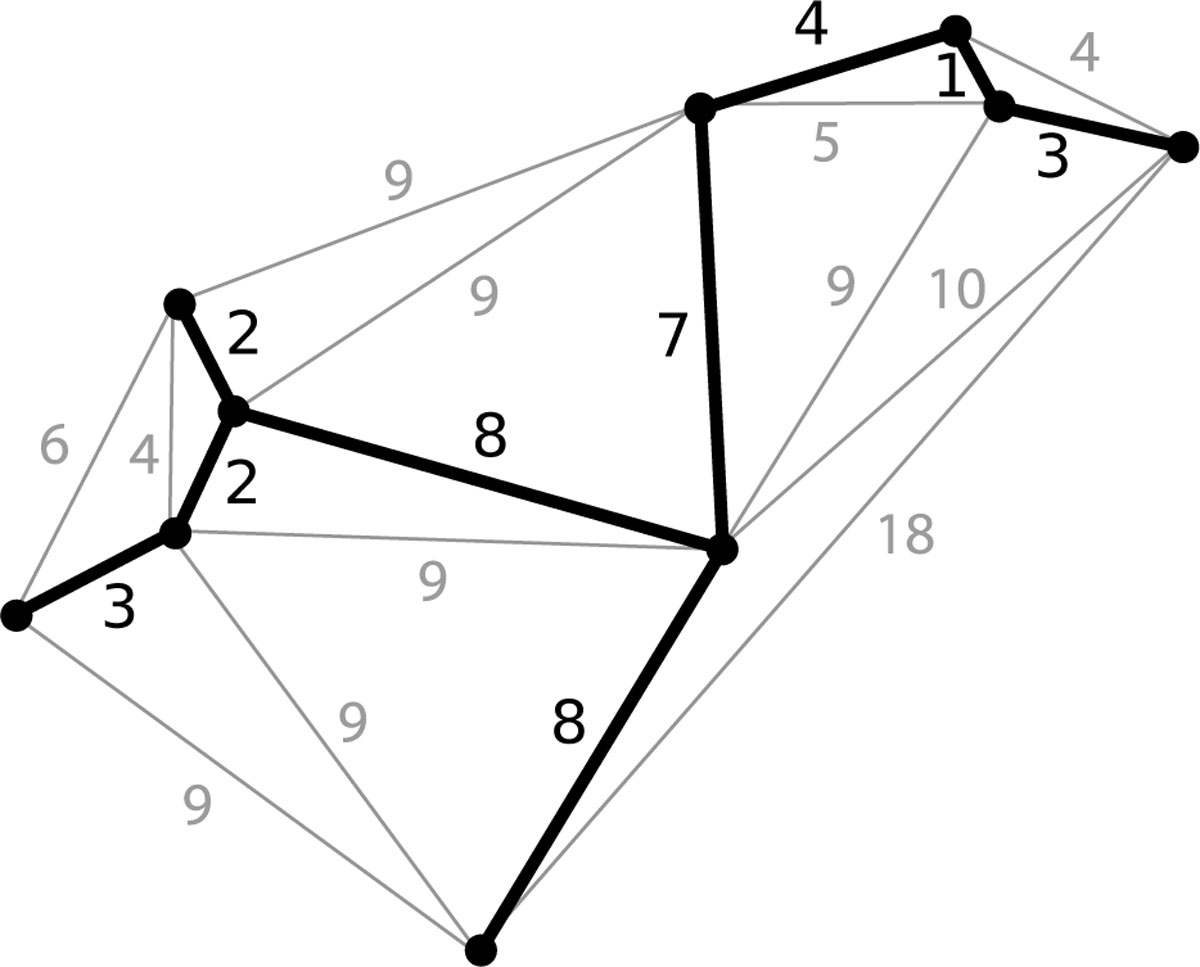
**A minimum spanning tree**.

Early approaches for inferring gene regulatory networks (GRNs) do not distinguish between direct regulatory interactions, such as the relationship between a transcription factor and a gene it promotes, and indirect relationships, such as co-regulation and co-expression. One such approach is inference of a minimum spanning tree (MST). MST algorithms identify the set of edges that connects all the nodes in a graph with minimum total weight. Figure 1 shows an example minimum spanning tree. While the edges MST algorithms include are among the strongest, they omit any edges not needed to span the graph. Network inference tools based on MSTs include Airnet [1] and Module Miner [2].

Newer methods go beyond inferring "relevance networks" to inferring direct relationships. Two information theory-based methods, ARACNe [3] and CLR [4] were among the first to distinguish direct interactions, such as those between genes and their transcription factors, from indirect ones, such as co-regulation and co-expression. This has also been done using maximum relevance-minimum redundancy feature selection [5], static and dynamic Bayesian networks [6,7], tree ensemble methods [8], and pathway-consistency algorithms using conditional dependencies [9].

Inferred GRNs resemble a chaotic "hairball" of nodes and edges. In this complex form, their rich information may not be very accessible. Inferring a simpler unit--the most likely chain of regulatory interactions that connects two genes of interest--may have advantages. A linear path may be surrounded by interactions that are also of interest, but it could capture the most important set of interactions in a comprehensible way. The path could be viewed in its network context by taking its union with other paths. If the path inference method can provide some means of sorting paths according to their likelihood of accuracy, this could be used to prioritize laboratory validation of interactions between genes of interest.

The probability of a path being composed of direct edges is the joint probability of directness of its component edges. The method presented in this paper exploits this fact to convert the pathway inference problem into a shortest-path problem, deriving the weight for each edge in the graph from its the estimated probability of directness. The weights are transformed such that finding the shortest path between two genes maximizes the product of the probabilities of its edges. The resulting path probabilities could theoretically be used to rank paths according to their likelihood.

The computational alternative to inferring paths directly is extracting paths from inferred networks. However, some network inference criteria may be maximized by omitting edges that would be needed in paths. And network inference may not lead to a systematic way of ranking extracted paths. With this in mind, two methods were chosen for comparison that offer interesting contrasts to the shortest-path method. One is an implementation of the ARACNe algorithm. The other is a basic MST method.

### Mutual information and the data processing inequality

Relationships between genes' expression levels can be quantified using a measure of dependency, such as Pearson's correlation, Spearman's correlation, Euclidean distance, or mutual information.

Butte and Kohane [10] first used mutual information (MI) to infer relevance networks. It has since become the dominant dependency measure in GRN inference. Unlike Pearson's correlation, MI captures non-linear dependence and makes no assumption about the form of the joint distribution. The MI of two random variables *X *and *Y *is defined as:

$$I\left(X;Y\right)=\iint p\left(x,y\right)\text{log}\left(\frac{p\left(x,y\right)}{p\left(x\right)p\left(y\right)}\right)dxdy$$

where *p(x) *and *p(y) *are the marginal probability distributions of *x *and *y*, and *p(x,y) *is the joint probability distribution. The actual MI of two random variables is an unknown parameter. Many methods have been proposed for estimating MI (see Walters-Williams and Li [11] for one review and comparison). Without strong distributional assumptions, no method has been shown to be optimal.

**Figure 2:**
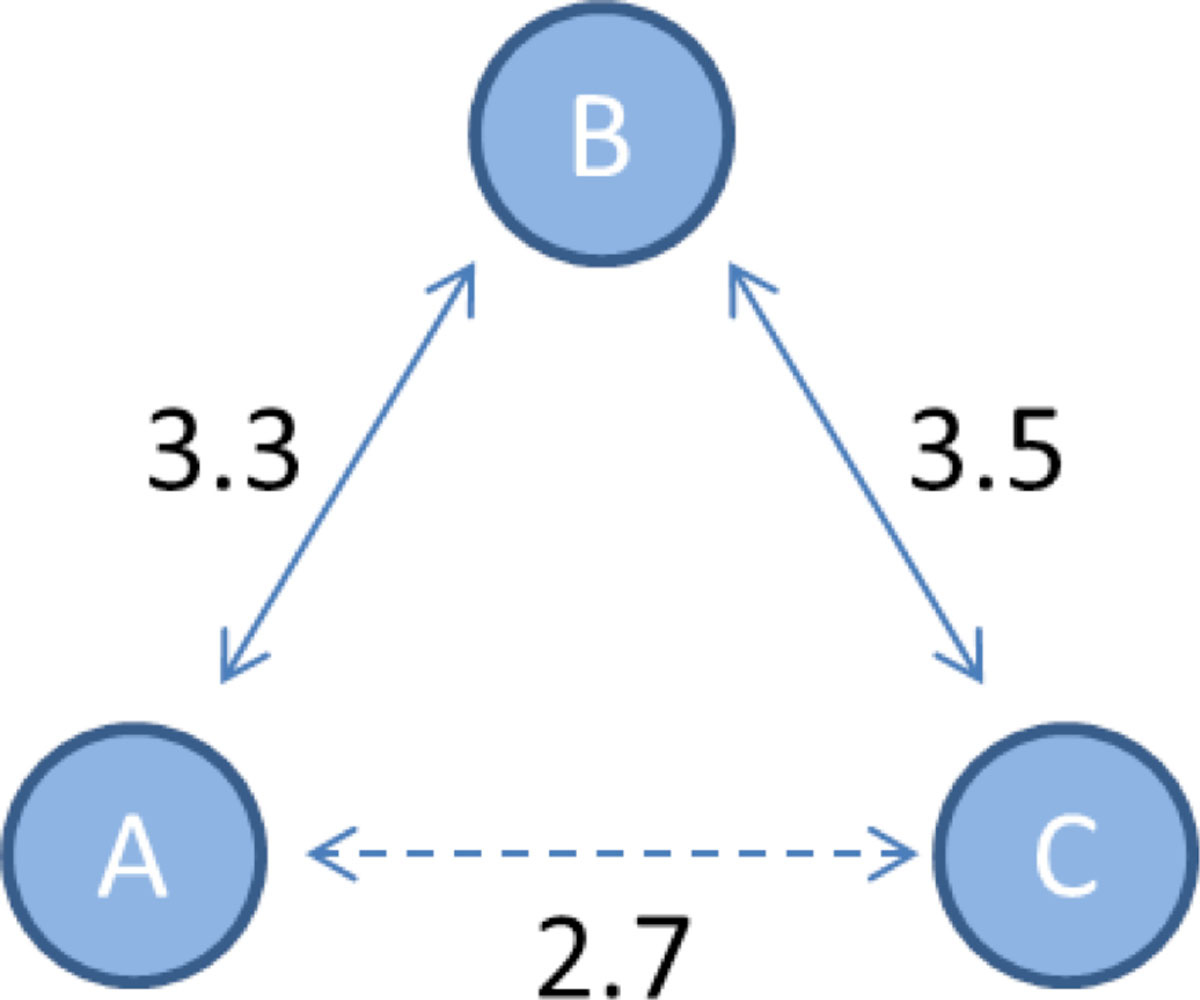
**DPI triplet comparison**.

Simple thresholding of MI estimates does not isolate direct relationships. However, directness can be inferred by comparison of MI values. One approach is based on the data processing inequality (DPI), whose use for GRN inference was pioneered by Basso et al.[3] in connection with the ARACNe algorithm. Take a trio of genes A, B, and C, where A influences C only through B (see Figure 2).

The DPI states that the information of the indirect relationship will be the least of the three. That is,

$$I\left(A;C\right)\leq min\left(I\left(A;B\right),I\left(B;C\right)\right).$$

The inequality can be taken to be strict in the context of biological processes. It can be shown that, if true MI values could be used rather than estimates, pruning edges based on the DPI would perfectly reproduce an acyclic graph.

Inference of directness based on the DPI is used in the shortest-path method as well, with the difference that a probability of directness is estimated for each edge, rather than a simple imputation of directness or indirectness. More details can be found in the Methods section.

## Results

In the network inference literature, as with other binary classification problems, performance is often measured by sensitivity (synonymous with recall) and specificity. These can be calculated from the number of true positives, false positives, true negatives, and false negatives.

$$sensitivity=\frac{tp}{tp+fn}\;\;\;\;\;\;\;\;\;\;\;\;\;\;\;\;\;\;\;\;\;\;\;specificity=\frac{tn}{tn+fn}$$

Precision is related to specificity but is more appropriate in the context of paths, since the number of true negatives (edges that were not included in the true path) dwarfs the number of edges in the path.

$$precision=\frac{tp}{tp+tp}$$

For this reason, precision and recall were chosen as measures of performance. Results are reported from paths longer than a single edge. This allows for comparison with the node-derived measures, which would be meaningless for paths of a single edge.

Most of the datasets used are from the DREAM3 *in silico *network inference challenge and were generated by the GeneNetWeaver package from 10 experimentally determined GRNs. There are 5 each (2 from *E. coli *and 3 from *S. cerevisiae*) of 10 and 50 genes. The other data set of 50 genes, from the R package Minet [12], was generated from a *S. cerevisiae *network by the SynTReN package. The network topology of the datasets differ. One characteristic expected to be of particular importance was the cyclicity of the network, here measured as the fraction of back edges encountered in a depth-first search.

**Figure 3:**
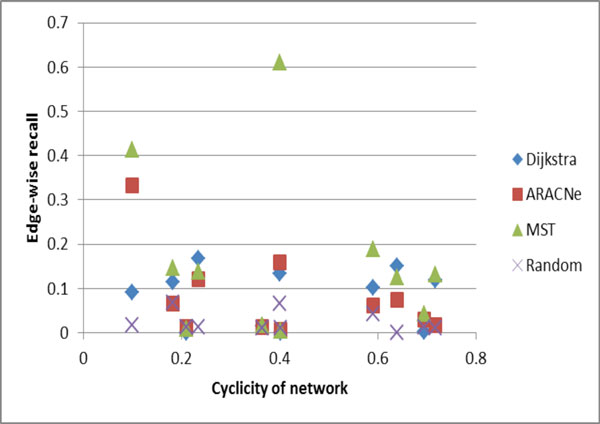
**Edge-wise recall by cyclicity**.

Absolute and relative performance varies across datasets. Figure 3 shows edge-wise recall when plotted against cyclicity across all datasets. Edge-wise precision was nearly identical to edge-wise recall. Node-wise recall and precision resemble their edge-wise counterparts but are generally higher, and the difference is greater for precision than for recall.

**Figure 4:**
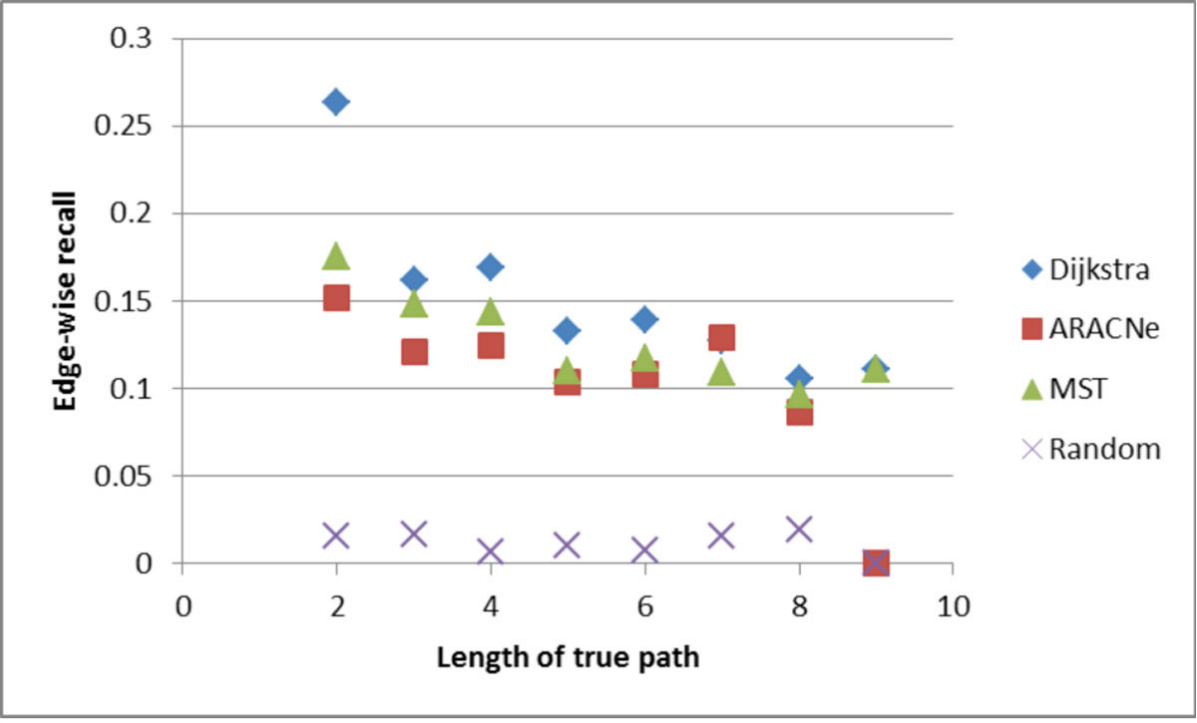
**Edge-wise recall by true path length**.

**Figure 5:**
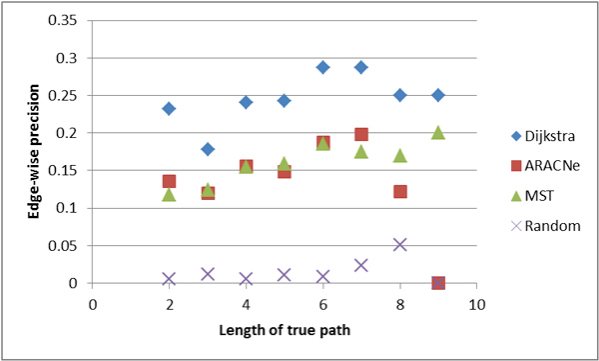
**Edge-wise precision by true path length**.

**Figure 6:**
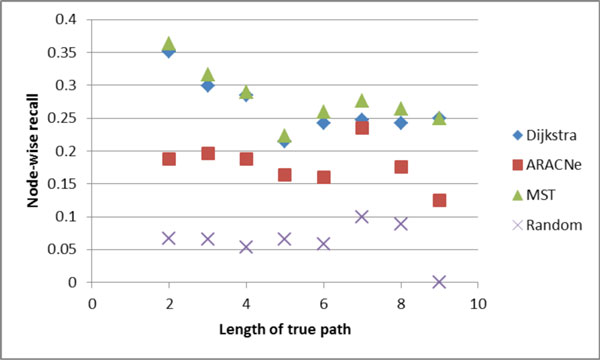
**Node-wise recall by true path length**.

**Figure 7:**
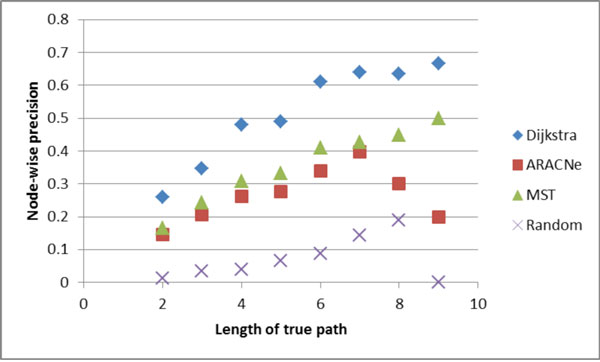
**Node-wise precision by true path length**.

Figures 4, 5, 6, 7 show the comparative performance of the different algorithms as the length of the true path increases. The results are from the Minet dataset. Edge-wise recall and node-wise recall both decrease as path length increases. Node-wise recall tends to be about twice as high as edge-wise recall. Edge-wise precision increases very slightly with path length. Node-wise precision also increases with path length, and the increase is greater than for edge-wise precision.

**Figure 8:**
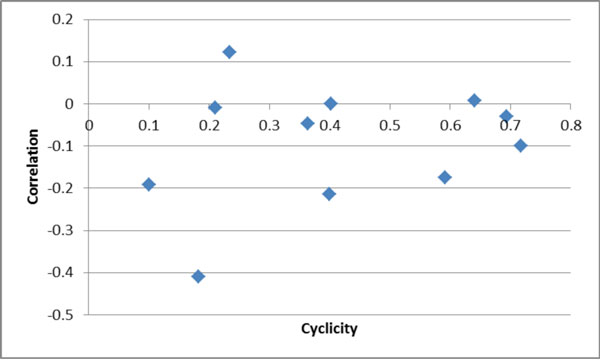
**Correlation of performance and path probability**.

As mentioned earlier, an additional goal of the direct path inference method is the ability to rank pathways according to their likelihood. If the derived probabilities of each edge's directness are accurate and independent, one would expect a strong relationship between the joint probability of the edges and the path's precision and recall. One of the simplest relationships that could exist between path probability and performance is a linear correlation. Figure 8 shows the correlation between path probability and the average of precision and recall as cyclicity increases. The correlation was weak and inconsistent. It appears to approach zero as cyclicity increases, and is most often negative.

### Improvements to other methods

The bootstrapping technique used to derive edge probabilities in the shortest-path method could also be used to increase the robustness of the mutual information estimates used by the other methods. The mean or median of the estimates from the bootstraps could be taken in place of the original estimate, reducing the effect of outliers. At the same time, this could result in some loss of sensitivity relative to using the full original dataset. It appears that both ARACNe and the minimum spanning tree algorithm tend to perform better using the mean of bootstrap estimates. The gains in robustness appear to outweigh the loss in sensitivity in most cases.

The negative log transformation used on edge probabilities in the shortest-path method could also be applied in connection with other methods that similarly seek to maximize some objective function using a minimization algorithm. For the MST method used in this paper, mutual information values are scaled by the maximum value and subtracted from one. Airnet uses a similar transformation, differing in that, since it uses Pearson's correlation, the values are already bounded between zero and one and do not need to be scaled by the maximum value. Using the negative log transformation for the MST achieved performance at least as good as that achieved using the subtract-from-one transformation, and negative log transformation may be more mathematically appropriate in some cases.

## Discussion

**Figure 9:**
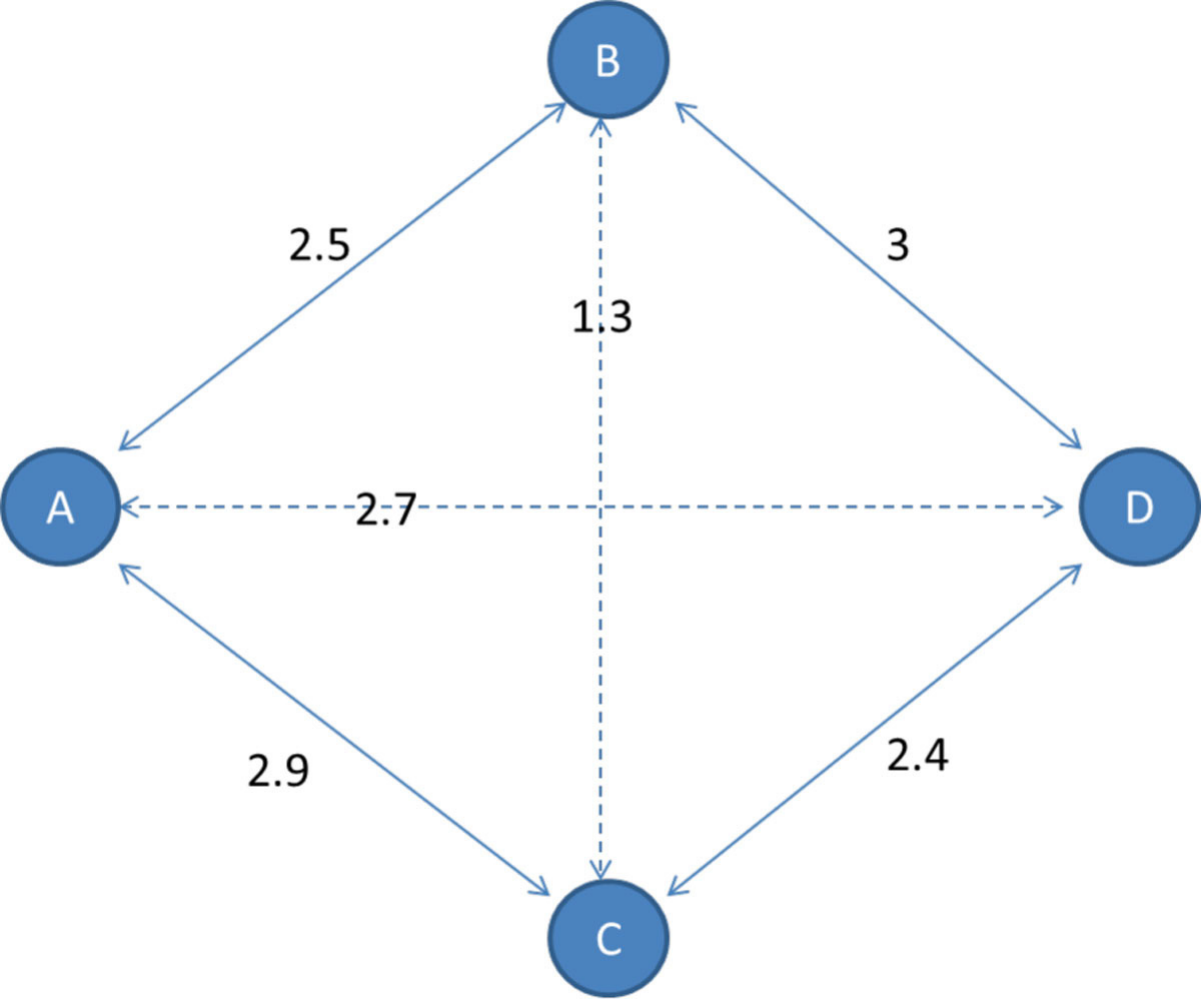
**Four-node cycle in which skipping could occur**.

The fact that node-wise performance (particularly precision) tends to exceed edge-wise performance, along with the tendency to find paths that are shorter than the true paths, could indicate skipping between correct nodes. This may occur in cycles, where a key assumption of the DPI does not hold--namely, that nodes are not related through multiple channels. If the sum of multiple relationships between two genes is strong enough, the algorithm may infer a direct connection with the node at the other end of the cycle. Figure 9 shows a four-node cycle where skipping would occur. In this case, true edges A-B and C-D would be pruned, and a direct interaction would be incorrectly inferred between A and D.

The inference of longer paths may be less sensitive to estimation errors than the inference of shorter paths. If a true edge in a path appears weak, strong edges in the path could compensate. However, detecting this effect would require better controls and a wider selection of networks than used in this study. While some of the results appear to confirm the hypothesis that inference of longer paths is more accurate, they should be interpreted with caution. The slight increase in edge-wise precision as true path length increases could reflect the fact that there are more ways of including correct edges in longer paths. The greater increase in node-wise precision with path length would be consistent with the hypothesis of shortcut errors; however, there are more ways to get correct nodes than correct edges for any maximum path length k (by a factor of (k-1)!), and random paths generated for comparison exhibit a similar upward trend.

The counterintuitive result that path probability is negatively correlated with precision and recall may relate to the kind of errors that occur. On average, inferred paths are shorter than the true paths. Each erroneous shorter path is chosen because it has higher joint probability than the correct path, whether due to incorrect calculation of edge probabilities or dependence between edge probabilities. When the most probable path between two nodes involves no shortcuts, it may tend to be longer and have lower probability. However, the correlation was weak and inconsistent, and could be an artifact of the way performance metrics were derived. In addition, nonlinear relationships could exist that would not be reflected by the correlation coefficient. Further investigation is needed to determine what relationships exist and how they could be used to rank paths.

## Conclusions

The performance of the shortest-path method appears comparable to the other two methods. However, the metrics used may not be optimal. Conditioning on true path length complicates generalizations about performance trends. Also, inferred paths are compared to the most direct path through the graph, ignoring valid but less direct paths. The measure of cyclicity used here (proportion of back edges) does not directly indicate the size and number of cycles. And the shortest-path method optimizes for finding the entire correct path, while the test metrics are based on finding any part of it. This may affect contrasts with MST algorithms, for example, which achieve low recall and high precision at the network level. MSTs are likely to do well in partial credit comparisons and poorly on all-or-nothing metrics.

Any method using MI is sensitive to the quality of MI estimation, which is difficult with the relatively small number of samples common in microarray data. The direct path inference method relies on estimation of the posterior distribution of MI, an even more difficult problem. Bayesian estimation could perform better with small sample sizes, and may enable incorporation of data about known regulatory interactions in a statistically sound way as priors.

Improvements could be made to the inference of directness in cycles. The type of skipping hypothesized here should occur most when the ratio of the number of paths between the end genes of the cycle and the length of the paths is high. However, relationships mislabeled by skipping are important ones despite being indirect.

There is at least one valid objection to the assumption of independent edge probabilities: they are based on triplet comparisons, and any two adjacent edges are jointly involved in one triplet comparison. The decisive comparison in classifying an edge as indirect will result in dependence between the edges in that triplet, particularly between edges whose MI values are closest. From a biological standpoint, expression levels of many genes are not independent (their dependency is what the MI describes), and the dependency between two genes may be related to the expression level of another gene (e.g. a co-activator or co-repressor), but it's unclear how dependence between the dependencies of genes would arise in a high enough proportion of regulatory scenarios to significantly affect this method's performance.

The code for this project can be found at http://dna.cs.byu.edu/shortest/, along with extensions enabling generation and use of larger datasets from GeneNetWeaver, comparison to the official implementation of ARACNe and its kernel MI estimator, and examination of the independence assumption through comparison of the empirical joint distribution of edges' probabilities of directness with the multiplicative joint distribution.

## Methods

### Path Inference

A path exists if each of its edges represents a direct regulatory relationship. The problem can be framed as finding the path that is most likely, given the data, to be composed entirely of direct edges. Let *RT *represent the set of all possible paths from *r *to *t*, and let *e_p _*be the set of edges in path *p*. Assuming the DPI holds, the edge between nodes *A *and *B *is direct if and only if it is not the least edge in any triplet comparison with any other node *C*. This condition will be abbreviated as *DPI*(*AB*):

$$DPI\left(AB\right):\Leftrightarrow \forall C:I\left(A,B\right)\geq \text{min}\left(I\left(A,C\right),I\left(B,C\right)\right)$$

Thus, the path is to be found with maximum probability of every edge being direct. In other words,

(1) $$\arg{\;\text{max}}_{p\in RT}P(\forall AB\in e_{p}:DPI(AB))$$

This requires finding the posterior joint probability of MI values. It can be approximated by bootstrapping. For each bootstrap round of MI estimation, each edge's directness according to the DPI is recorded. Its probability of directness is taken to be the proportion of bootstraps in which it is considered direct.

The probability of a path is the joint probability of its edges. The number of possible paths in a complete graph of *n *nodes is exponential, and the number of paths under some length threshold *k *(e.g. a limit of biological plausibility) is *O*(*n*^(*k-1*)^). Computing probabilities for all paths is infeasible for large graphs. However, if edges' being direct are independent events, the joint probability of edges in a path is equal to the product of edge probabilities. Then (1) can be expressed as

(2) $$\text{arg}\;\text{max}\underset{AB\in e_{p}}{\prod }P\left(DPI\left(AB\right)\right)$$

Since the logarithm is a monotonic increasing transformation, order is preserved when (2) is expressed as

(3) $$\text{arg}\;\text{max}\;\text{log}\left(\underset{AB\in e_{p}}{\prod }P\left(DPI\left(AB\right)\right)\right)=\text{arg}\;\text{max}\underset{AB\in e_{p}}{\sum }\text{log}\left(P\left(DPI\left(AB\right)\right)\right)$$

Which in turn is equivalent to

(4) $$\text{arg}\;\text{min}\underset{AB\in e_{p}}{\sum }-\text{log}\left(P\left(DPI\left(AB\right)\right)\right)$$

This reduces pathway inference to a shortest-path problem, using as edge weights the negative log of the probability that each edge is direct. Since the transformed edge weights are positive, Dijkstra's algorithm can be used to find the shortest path between a given root node and all other nodes in a graph. Using priority queues, Dijkstra's algorithm is of *O*(*E *+ *nlog*(*n*)) complexity, where *E *is the number of edges and *n *is the number of nodes. Dijkstra's algorithm was used previously by Gebert et al. [13] in a differential equation framework to explore regulatory network connectivity and identify influential genes.

For the baseline random paths set, one random path was generated for each combination of root and target nodes, of a random length no greater than that of the longest true path in the true network.

The ARACNe method referred to in this paper is an independent implementation of the algorithm described in Margolin et al. [14]. The ARACNe algorithm applies a significance threshold (derived by a permutation test) to MI estimates and prunes edges according to the DPI with a tolerance margin. For path inference, it performed best with a stringent tolerance margin and a permissive significance threshold.

The MST network inference method applied Prim's algorithm to pairwise transformed MI. In order for the minimum spanning criterion to optimize the strength of included edges, each MI value was transformed by scaling by the maximum value in the dataset and then subtracting from one. Airnet applies a similar transformation using Pearson's correlation.

For both the ARACNe and MST methods, Dijkstra's algorithm was used to extract shortest paths from the inferred network, with each edge in the network having unit weight.

## Competing interests

The authors declare that they have no competing interests.

## Authors' contributions

JDJ conceived the basic idea, led the project, and performed the implementation and testing. DMJ provided crucial mathematical insights, experimental suggestions, and proofreading. MJC and QOS provided important resources, feedback, and instructions on certain aspects of the implementation.

## Declarations

This research was funded in part by an anonymous donor in connection with the Robert K. Thomas scholarship.

This article has been published as part of BMC Bioinformatics Volume 14 Supplement 13, 2013: Selected articles from the 9th Annual Biotechnology and Bioinformatics Symposium (BIOT 2012). The full contents of the supplement are available online at http://www.biomedcentral.com/bmcbioinformatics/supplements/14/S13
